# Adherence to Secondary Prevention and Influential Factors in Individuals with Coronary Angioplasty

**DOI:** 10.17533/udea.iee.v38n3e05

**Published:** 2020-11-11

**Authors:** Vanesa Henao López, Zaider Triviño Vargas

**Affiliations:** 1 Nurse, Masters. Nurse in the Adult Echocardiography service at Clínica Cardio VID. Medellín, Colombia. Email: vanesahl7@hotmail.com Clínica Cardio VID Medellín Colombia vanesahl7@hotmail.com; 2 Nurse, Ph.D. Full Professor, Universidad del Valle. Cali, Colombia. Email: Zaider.trivino@correounivalle.edu.co Universidad del Valle Universidad del Valle Cali Colombia Zaider.trivino@correounivalle.edu.co

**Keywords:** treatment adherence and compliance, secondary prevention, self care, acute coronary syndrome, angioplasty, estudios transversales, cumplimiento y adherencia al tratamiento, prevención secundaria, autocuidado, síndrome coronario agudo, angioplastia, cross-sectional studies, cooperação e adesão ao tratamento, prevenção secundaria, autocuidado, síndrome coronariana aguda, angioplastia, estudos transversais

## Abstract

**Objective.:**

To explore the relation between adherence to secondary prevention and factors that influence on said adherence in people with acute coronary syndrome, who underwent percutaneous coronary angioplasty in a clinic in Medellín.

**Methods.:**

Cross-sectional study on a random sample of 128 volunteer patients. A questionnaire was used for sociodemographic variables, the “Scale to measure therapeutic adherence for patients with chronic diseases, based on explicit behaviors” by Trujano, Vega, and Nava and the “Instrument to evaluate adherence by patients according to influential cardiovascular risk factors” validated by Consuelo Ortiz.

**Results.:**

Socioeconomic factors influenced in very low manner on the adherence to secondary prevention; factors related with the therapy did so moderately and patient factors influenced in low manner. No relation was found between the health provider factor and said adherence.

**Conclusion.:**

Factors exist that influence in a lesser or higher measure on adherence to secondary prevention and which must be recognized in people with coronary angioplasty to design strategies to improve this aspect of self-care.

## Resumen

**Objetivo.:**

Explorar la relación entre la adherencia a la prevención secundaria y los factores que influyen en dicha adherencia en personas con Síndrome Coronario Agudo, a quienes se les realizó angioplastia coronaria percutánea en una clínica de Medellín.

**Métodos.:**

Estudio de corte transversal en una muestra aleatoria de 128 pacientes voluntarios. Se utilizó un cuestionario para variables sociodemográficas, la “Escala para medir la adherencia terapéutica para pacientes con enfermedades crónicas, basada en comportamientos explícitos” de Trujano, Vega y Nava y el “Instrumento para evaluar la adherencia de los pacientes según factores influyentes de riesgo cardiovascular” validado por Consuelo Ortiz.

**Resultados.:**

Los factores socioeconómicos influyeron de manera muy baja en la adherencia a la prevención secundaria, los factores relacionados con la terapia lo hicieron en forma moderada y los factores del paciente influyeron de manera baja. No se encontró relación entre el factor del proveedor de salud y dicha adherencia.

**Conclusión.:**

Existen factores que influyen en menor o mayor medida en la adherencia a la prevención secundaria y que deben ser reconocidos en las personas con angioplastia coronaria para que se diseñen estrategias para el mejoramiento de este aspecto del autocuidado.

## Resumo

**Objetivo.:**

Explorar a relação entre a adesão à prevenção secundária e os fatores que influenciam essa adesão em pessoas com Síndrome Coronariana Aguda, submetidas a angioplastia coronária percutânea em uma clínica de Medellín (Colômbia).

**Métodos.:**

Estudo transversal em amostra aleatória de 128 pacientes voluntários. Foi utilizado um questionário para variáveis ​​sócio-demográficas, a “Escala para medir a adesão terapêutica para pacientes com doenças crônicas, baseada em comportamentos explícitos” de Trujano, Vega e Nava, e o “Instrumento para avaliar a adesão do paciente de acordo com fatores de risco cardiovascular influenciadores” validado por Consuelo Ortiz.

**Resultados.:**

Os fatores socioeconômicos tiveram uma influência muito baixa na adesão à prevenção secundária, os fatores relacionados à terapia tiveram uma influência moderada e os fatores do paciente tiveram uma influência baixa. Não foi encontrada relação entre o fator provedor de saúde e a referida adesão.

**Conclusão.:**

Existem fatores que influenciam em menor ou maior grau a adesão à prevenção secundária e que devem ser reconhecidos em pessoas com angioplastia coronariana para que estratégias possam ser traçadas para melhorar esse aspecto do autocuidado.

## Introduction

Due to coronary cardiopathy, 7.4-million people died in 2015 according to reports by the World Health Organization (WHO).(1) Since 2004, registries indicate that in Medellín this disease is the principal cause of mortality in people over 45 years of age.(2) For patients with Acute Coronary Syndrome (ACS) it is recommended that besides an interventionist management, secondary prevention(3) be applied. Investigations exist aimed at demonstrating the benefits of this, which must be considered by nursing and other health professions for their application; one of these investigations is the study PRESENTE(4) study conducted with 4174 post-infarction patients, showing effectiveness in secondary prevention regarding mortality and re-infarction outcomes with pharmacological treatment, incorporation of patients to the cardiac rehabilitation program, having a beneficial early effect on endothelial dysfunction, as well as diminished adverse effects. However, many times, the results are not those expected due largely to non-adherence by the patient, which is influenced by various factors (socioeconomic, related with the disease, with the treatment, with the health system, and with the patient) that can be favorable or not in this process. In addition, we must recognize that the patient is not the only one responsible for the adherence, but also the health staff, the health system, and regulatory organisms.(5) This is why it is useful to consider the Self-care Deficit Theory by Dorothea Orem, which declares that nursing must generate activities for patients to promote and maintain life, health, and wellbeing;(6) thereby, working for patients and their families to learn what ACS is, promote adherence to secondary prevention - bearing in mind the influential factors, and, over time, evaluate the efficacy of the work done.

This research assumed the definition of therapeutic adherence adopted by the WHO, which states that it is “the degree to which a person’s behavior -taking the medication, following a feeding regime, and executing lifestyle changes-corresponds with the agreed recommendations by a healthcare provider.”(7) Thus, it is considered that secondary prevention requires patients to have therapeutic adherence. The research sought to broaden knowledge on this theme by exploring the relationship between adherence to secondary prevention and the factors influencing said adherence in people with ACS, who underwent percutaneous coronary angioplasty (ACP) in a clinic in Medellín.

## Methods

This was a cross-sectional and correlational quantitative study, with a sample of 128 volunteer patients diagnosed with ACS and who underwent ACP in a clinic in Medellín between October 2017 and February 2018. Data was collected from three to four months after the event. Participants were selected by performing simple random probabilistic sampling, with 95% reliability and 5% estimated error. The study had the data base of 189 patients and 128 were selected. When gathering the information, it was found that six patients had died, which is why the invitation calls to participate in the research continued until completing the sample.

Eligibility criteria were: women and men > 18 years of age admitted with ACS diagnosis, intervened with percutaneous coronary angioplasty in the clinic, residents in the urban area of Medellín (Colombia). The study excluded those who besides ACP received surgical revascularization. To collect information, initially a written consent was created, but because information was collected via telephone, a call protocol was implemented that followed rigorously a check list to provide clear information to participants fulfilling the ethical criteria, to obtain the verbal informed consent and request data about (i) sociodemographic variables (age, sex, educational level, work situation) and health aspects (weight, height, dependence on care by others, suffering from other diseases), (ii) *Scale of therapeutic adherence for patients with chronic diseases*, based on explicit behaviors created by Trujano, Vega and Nava;(8) this scale has a Cronbach’s alpha value of 0.91 and the factorial analysis found it has three factors with seven items each: control of medication and food intake, medical behavioral monitoring, and self-efficacy. This scale has 21 items, whose score ranges from 0 to 100. The score is interpreted, thus: 0 - 33 points, low adherence; 34 - 67 points, moderate adherence; 68 - 100 points, high adherence, (iii) *Instrument to evaluate adherence to treatments in patients with cardiovascular risk factors* validated by Consuelo Ortiz.(9) This instrument reduced from 53 to 24 the items from the scale *Factors influencing on adherence to pharmacological and non-pharmacological treatment in patients with risk factors of cardiovascular disease* by Bonilla and De Reales.(10) This shortened version of the original instrument has a Cronbach’s alpha of 0.60. The factors are socioeconomic, those related with the provider: system and health staff, those related with the therapy and those related with the patient. The factor related with the disease characteristics was included in the items of the four factors evaluated. The response options were never, sometimes, or always. The score interpretation is <60% cannot respond with adherence behavior, 60%-79% at risk of not developing adherence behavior, 80%-100% advantage for adherence.

The information was collected through *Google Form* and the SPSS program, version 24, was used for its analysis. Sociodemographic data were analyzed to show a description of the study participants. The qualitative variables used measures of central tendency and dispersion. A bivariate analysis used the Shapiro-Wilk test to check the assumption of normality of the scores of the scales. Upon confirming the assumption, Student’s t test and ANOVA were used to identify if influential factors are related or not with therapeutic adherence. Finally, Pearson’s correlation coefficient was used to determine the relation between the scores from the scale of influential factors and scores from the scale of therapeutic adherence. A 5% significance level was used for all the statistical tests.

The Human Ethics Committee of the Faculty of Health of the Universidad del Valle endorsed the study and the clinic’s Research Ethics Committee authorized it.

## Results

It can be noted in Table 1 that the general characteristics prevailing in the study sample were: mean age of 65.12 years, 65.6% were of male sex, 57.8% were married, 63.2% had secondary or higher education, 26.1% was retired, 81.5% belong to socioeconomic levels 1 to 3, 61% live in their own home, and 89.1% receive economic monthly income of less than two Legal Minimum Wages (LMW). It was found that 91.3% of these patients live with at least one person, 68.5% are not dependent on care, 84.3% suffer from other diseases (arterial hypertension, diabetes mellitus, and hypothyroidism), 38.1% have had cardiology control in another institution, and 91% have self-perception of adherence between good and excellent, and mean BMI was 25.07.

**Table 1:** Sociodemographic characteristics and health aspects of 128 patients with acute coronary syndrome with percutaneous coronary angioplasty

Variables	Values
Age; mean ±SD (Minimum - Maximum)	65.1±10.6 (35-90)
BMI; mean ±SD (Minimum - Maximum)	25.1±3.7 (15.8-35.8)
Sex: n(%)	
Female	44 (34.4)
Male	84 (65.6)
Marital status; *n* (%)	
Single	11 (8.6)
Separated	10 (7.8)
Widowed	17 (13.3)
Married	74 (57.8)
Divorced	5 (3.9)
Common law	11 (8.6)
Educational level; *n* (%)	
Primary	46 (36.8)
Secondary	35 (28.0)
Technical/Technological	15 (12.0)
University	11 (8.8)
Graduate	1 (0.8)
Other	17 (13.6)
Home ownership; *n* (%)	
Rented	22 (17.9)
Own	75 (61.0)
Family	24 (19.5)
Other	2 (1.6)
Socioeconomic level; *n* (%)	
1 to 3	97 (81.5)
4 to 5	18 (15.1)
6	4 (3.4)
Work situation; *n* (%)	
Full-time job	15 (13.0)
Part-time job	2 (1.7)
Unemployed in search of a job	8 (7.0)
Unemployed, not looking for work	26 (22.6)
Independent	21 (18.3)
Retired (work)	30 (26.1)
Pensioned (disease, family)	13 (11.3)
Monthly economic income in Legal Minimum Wages (LMW)*; n (%)	
Less than the minimum wage	15 (27.3)
1 to 2	35 (63.6)
3 or more	5 (9)
People living with; *n* (%)	
0	12 (9.7)
1	22 (17.7)
2 to 3	51 (41.1)
4 to 5	29 (23.4)
6 or more	10 (8.1)
Dependent on care from other people; *n* (%)	
No	87 (68.5)
Totally	24 (18.9)
Partially	16 (12.6)
Suffers from other diseases; *n* (%)	107 (84.3)
Has had cardiology control in another institution; *n* (%)	48 (38.1)
Perception of the adherence process; *n* (%)	
Excellent	71 (55.5)
Good	48 (37.5)
Regular	8 (6.3)
Bad	1 (0.8)

* 1 LMW for 2018 = $781 242 Colombian pesos. Note: 1 US Dollar = 3623 pesos

With respect to the results in the scale of therapeutic adherence for patients with chronic diseases based on explicit behaviors, it was found that patients in general have high adherence with 96.1%, and - likewise - in each of the three factors, medical behavioral monitoring obtained a score of 97.7%, followed by the control on the intake of food and medications with 91.4%, and self-efficacy with 89.1%.

Table 2 displays the mean scores and standard deviations of the factors from the scale of therapeutic adherence in function of some aspects related with health. It is highlighted that dependence on a caregiver is a factor related significantly with adherence to medical behavioral monitoring and self-efficacy, although not positively, that is, that those patients who depend totally on care from another person have lower medical behavioral monitoring than those who do not depend or depend partially on another person. It was also identified that patients attending other institutions for cardiology control have lower self-efficacy compared with patients who attended the institution where the study was conducted, a finding that was also significant. The patient’s perception on the adherence process is significantly related with the three factors of therapeutic adherence. The other items studied had no significant relation.

**Table 2:** Factors of therapeutic adherence in function of some factor aspects related with hea**lth**

Aspects	Categories	Control of food and medication intake	Control of food and medication intake	Medical behavioral monitoring	Medical behavioral monitoring	Self-efficacy	Self-efficacy
		Mean ±SD	*p*-value*	Mean ±SD	*p*-value*	Mean ±SD	*p*-value*
Dependence on care from other people	No	89.5±13.6	0.409	97.0±7.0	0.008	90.7±13.1	0.008
Dependence on care from other people	Totally	84.9±19.7		90.1±16.2		80.1±20.9	
Dependence on care from other people	Partially	88.4±12.4		95.4±6.2		86.1±12.7	
Has had cardiology control in another institution	No	90.2±13.9	0.077	96.4±9.3	0.196	90.7±14.7	0.018
Has had cardiology control in another institution	Yes	85.4±15.9		94.0±10.2		84.0±15.7	
Perception on the adherence process	Excellent	91.3±11.5	<0.0001	96.6±7.8	0.047	90.5±12.2	0.009
Perception on the adherence process	Good	88.0±14.1		95.2±11.2		87.6±15.9	
Perception on the adherence process	Regular	66.8±25.6		87.5±12.1		71.6±25.8	
Perception on the adherence process	Bad	92.9		85.7		85.7	

* Student’s t test and ANOVA test

Table 3 shows the Pearson correlations among the scores from the scales of influential factors and scores from the scale of therapeutic adherence. The following mentions the statistically significant correlations:

Regarding socioeconomic factors, a low positive relation was found with adherence to intake of food and medications, and a very low positive relation, which indicates that better socioeconomic aspects are related with adequate administration of medications and diet, and with better therapeutic adherence, although to a lesser measure. With respect to factors related with therapy, this yielded a moderate positive relation with adherence to medication intake control, medical behavioral monitoring, self-efficacy, and to therapeutic adherence in general. Hence, better aspects of therapy improve adherence to the consumption of appropriate foods; also, correct administration of medications favors effective behaviors to follow medical recommendations and the self-efficacy; that is, it leads to better therapeutic adherence.

With respect to patient-related factors, a low positive relation was noted with adherence to medication intake control, to self-efficacy, and to therapeutic adherence in general; and a very low positive relation with medical behavioral monitoring. Thereby, better aspects related with the patient favor an appropriate diet and administration of medications, the patient’s self-efficacy, and therapeutic adherence; and, thus, favors effective health care behaviors, according to medical indications, although in lower measure. In the other correlations, no statistically significant results were found.

**Table 3:** Relation between associated factors and therapeutic adherence

**Adherence factors**	Factors influencing on adherence	Factors influencing on adherence	Factors influencing on adherence	Factors influencing on adherence
	Socioeconomic	Systems and health staff	Therapy	Patient
Intake of food and medications	0.271^*^	0.098	0.536^*^	0.237^*^
Medical behavioral monitoring	0.047	0.047	0.528^*^	0.187^*^
Self-efficacy	0.083	0.038	0.444^*^	0.226^*^
Therapeutic adherence	0.179*	0.078	0.619*	0.274*

* *p*-value <0.05

## Discussion

The research had a higher proportion of men with el 65.6%, which coincides with other studies, like that reported by Chavarriaga *et al.,*(11) and by Ferreira-González,(12) who mentions in “Epidemiology of coronary disease” that the prevalence of heart conditions has shown strong male dominance. Regarding age, the mean was 65.2 years, coinciding with data published by the American Heart Association(13) where the mean age of the first heart attack is 65.3 years for men, and 71.8 years for women.

The population studied has a protection factor related with the housing variable, which in highest proportion was owned; adding to this the fact that 58% of the patients correspond to socioeconomic levels 3 and above; however, it is concerning that the lowest socioeconomic levels of the population show a high percentage (42%), comparable numbers of the data registered by the Mayor’s Office of Medellín,(2) who report that 21.9% are in socioeconomic level 1 and 48% in level 2; these patients need to receive special care, given that for the most part they only have income lower than two minimum wages, which makes one think that this fact implies difficulties to care for basic needs, like access to an optimal nutrition, affording medications in cases where the insurance carrier does not provide them, and attending medical control appointments, among others. This aspect must be kept in mind to provide other alternatives. 

With respect to the company the patients keep, of any type, most of the study subjects are accompanied and have family support; however, no necessarily do they always receive care from their relatives. Relating this finding with la Self-care Theory by Orem, this result gives a perspective that patients are self-care agents, that is, “deciding what they can and should do for their health and wellbeing over time”;(14) it is expected that the self-care capacity of patients must have improved after discharge and that according to the individual condition of each patient does not depend on others to adhere to secondary prevention; it is highly likely that this fact demonstrates the aid and education provided by nursing to patients regarding their self-care.

It is important to highlight that the cardiology control appointment after discharge depends on the insurance carrier, which decides if the patient continues the controls in the clinic or in another institution. It was evidenced that those attending another institution see adherence affected in the self-efficacy factor due to the change of institutions that possibly presents differences in their management protocols; this is important because some administrative policies could generate discomfort in patients need continuity with the treating cardiologist, given that said cardiologist already knows their health-disease process; additionally, as demonstrated, this situation affects the adherence of the individuals in the present study, specifically in the self-efficacy factor; in this regard, the WHO(7) confirms that when care is received by the same professional, as time goes by patients demonstrate better therapeutic adherence.

Likewise, in the variable asking if the patient depended on the care from another person, it was detected that said dependence affects the factors: medical-behavioral monitoring and self-efficacy. This gains importance because the patient’s caregiver (partial or primary) fulfils an indispensable role on adherence; however, upon evaluating the items from the scale, comprising said factors, it is easy to note that there are items in which the caregiver cannot fulfil this role, like, for example, be able to watch for symptoms, feel confident if the doctor demonstrates knowing the disease or feel sure of the disease he has and stick to the treatment, given that these are very personal functions. In addition, the other items from these factors can also be affected with overburden implied by caring for another person; that is, being attentive of the individual activities and the activities of the other, like administering medications punctually, taking the patient to scheduled exams and attending medical appointments, as expressed by Carreño *et al*.,(15) this overburden implies “physical fatigue, which has repercussions in the decrease of their daily activities due to lack of energy and in the alteration of their cognitive functions”; finally, by self-efficacy being a behavior influenced by the expectation of success, it becomes something personal and is affected when depending on another.

The study acknowledges that participating patients had high adherence with 96.1%, which does not coincide with that reported by other studies where adherence is lower; however, the study by Rojas and Flórez(16) with 180 patients with acute myocardial infarction found that those with greater adherence were patients with less than two years (58%) or more than five years (63%) after the event and merely 2% of the patients who had between six months and one year were classified as adherent. Hence, it is necessary to bear in mind that this study was conducted only between three and four months after the medical discharge and the fact that the results obtained do not coincide with other investigations reporting deficient adherence to pharmacological and non-pharmacological treatment, the following two reasons are submitted to discussion: the first are the differences in the sociodemographic characteristics and size of the sample implemented, which can affect the results; but above all, the second reason, considering that the factors related with adherence vary over the time transpired since the diagnosis, and with it the impact upon said adherence. The adherence process is a complex process that can depend even on the moment patients are going through, given that the closeness of this experience with the Acute Coronary Syndrome can sensitize and motivate them to be adherent; however, the passage of time and the sensation of health may cause patients not to be rigorous with the treatment and falter; therefore, it is submitted to discussion that this study teaches us the importance of providing continuity and paying attention to patient’s adherence over time, being an important task of nursing professionals to motivate patients to not abandon the treatment and maintain adherent; thus, following the recommendations by Orem,(17) who states that “self-care must be applied deliberately and continuously over time, always in correspondence with the regulation needs individuals have in their stages of growth and development, states of health, health characteristics or specific developmental phases, and environmental factors”.

The purpose of this study sought to determine the relation between influential factors and adherence to secondary prevention, which is measured through Pearson’s correlation and through this it was evidenced that: the highest relation that took place is that between the factors related with the therapy and adherence, with this being a moderate positive relation; that is that better aspects related with the patient’s knowledge about taking medications, distances to attend control appointments, customs on foods and exercise, occupations in and out of the home, lead to better patient adherence regarding medication and food intake, medical behavioral monitoring, and self-efficacy. It must be highlighted that within this aspect, nursing work is implicit, given that the education it provides is aimed at sensitizing the patient with respect to customs difficult to change, strengthening knowledge about medications, emphasizing on the importance of not abandoning the treatment and on seeking alternatives so they won’t forget to take the medication. Likewise, Orem(6) - through the educational support system mentioned in the theory - considers that people need to guided and educated to achieve self-care and the nurse plays a fundamental role.

Thereafter, we find the relation between patient-related factors and adherence, with this being a low positive relation with each of the adherence factors, except for the relation with the medical behavioral monitoring that was very low; that is that the less convinced patients are regarding the benefits of the treatment, the higher their interest in knowing about their health and how to care for themselves, and their conviction about the responsibility of health care, the greater will be the patient’s adherence with respect to medication and food intake, medical behavioral monitoring, and self-efficacy. In this regard, in the research by Moral and Cerda(18) in patients with diabetes, they coincide in that higher perception of improvement and control of the disease means less problems of adherence to treatment.

With respect to the relation between socioeconomic factors and adherence, only a low positive relation was found between socioeconomic factors and medication and food intake; in other words, having the economic availability to meet the basic needs, the capacity to afford medications, to travel to the place of consultation, read written information about the disease, having the money to modify the diet, as well as having support from the family and close friends, favor adherence to ingest recommended foods and take the medications prescribed. In this sense, the study by Chacon *et al.,*(19) in Chile with hypertensive patients evidenced the relation between adherence to the treatment and the socioeconomic factor, which is logical, given that secondary prevention demands a strict diet (diminished consumption of salt, sugar, carbohydrates, and fats) and if the patient has economic resources and family and social support it will be easy to access this type of diet. Similarly, if the insurance carrier does not provide the medication prescribed and the patient has the economic capacity to acquire it, optimal adherence could be achieved. Although it was not possible to demonstrate existence of a significant relation between socioeconomic factors and the other adherence factors, a very low positive relation does exist between socioeconomic aspects and therapeutic adherence, which could be explained by the fact that for patients to have adequate adherence, the socioeconomic aspects do not influence so much to achieve medical-behavioral monitoring and self-efficacy.

It was not possible to demonstrate the presence of relation between the factors related with the provider (systems and health staff) and adherence, which does not coincide with that mentioned by the WHO and other investigations that report the influence of the provider on adherence, including the work by governmental entities, insurance carriers, and the health staff. In the research by Ortega and Vargas,(20) this factor showed higher difference among the different groups of situation of adherence, considering that the relation existing between patients and the health provider is quite important on the degree of adherence. With respect to this, the WHO(7) is emphatic in stating that the health care system impacts potentially on the patients’ adherence behavior. In factors related with the provider, communication is highlighted between patients and the health staff, which is essential to achieve the patients’ motivation and participation in their health process; moreover the WHO(7) encourages communication that keeps patients participating in health care, considering it a simple and economic strategy that improves therapeutic adherence.

This research found differences in self-efficacy among patients who continued controls with cardiology in the clinic where this study was conducted, and those who continued in another institution; thereby, in this case the health staff did influence on the self-efficacy of patients. This does not mean a contradiction in the findings, given that it must be kept in mind that the finding of no positive correlation existing between the provider and self-efficacy was performed with all the patients grouped, that is, patients who attended another institution and those who continued controls with cardiology in the clinic, hence the results change. Upon not evidencing positive correlation between the factors related with the provider and the factors integrating adherence, both the institution where the study was conducted and the insurance carriers must know these findings for visibility implementation regarding the relevance of the adequacy of health care equipment and systems in their entirety and context. Likewise, it is fundamental for nursing to know of the factors influencing on adherence by each patient, to plan and carry out the educational intervention, to generate positive impact on patients and for this to be reflected on their adherence.

Knowing the influence exerted by socioeconomic factors, provider (system and health staff), therapy, patients, and disease on people with ACS, the work nursing must perform favors in boosting adherence to secondary prevention. The conclusion of this study is that, although each person’s process is different, factors exist that influence to a lesser or greater extent and which must be recognized by the health staff to take action in said regard. The work performed must be constant to guarantee the individuals do not falter in the adherence with the passage of time.

For this study, the principal limitation was achieving personalized communication with the patients, given that the purpose implied that the information would be obtained after discharge, which was complicated to make them return to the institution to apply the evaluation instruments; therefore, the call protocol was followed, which had been applied in prior investigations in the clinic and had been successful.
